# FACTORS THAT CONTRIBUTE TO FALLS AMONG AL RESIDENTS

**DOI:** 10.1093/geroni/igz038.879

**Published:** 2019-11-08

**Authors:** Marie Boltz

**Affiliations:** Pennsylvania State University, University Park, Pennsylvania, United States

## Abstract

Many nurses, patients, and families continue to believe that physical activity increases the risk of falling. The purpose of this study was to test the hypothesis that residents who are exposed to Function Focused Care for Assisted Living (FFC-AL-EIT) and engage in moderate levels of physical activity would not be more likely to fall. This was a secondary data analysis using data from the first two cohorts of the FFC-AL-EIT study. The study included 508 residents the majority of whom were female (70%), white (97%), with a mean age of 87.72 (SD=7.47). Those who engaged in more moderate intensity physical activity were 1% less likely to fall (b = -.01, Wald =6.13, p =.01). There was no association between exposure to function focused care and falling (Beta =.41, Wald =2.35, p=.13). Further, engaging in moderate level physical activity was noted to be slightly protective of falling.

